# High-throughput sequencing-based assembly of chloroplast genomes of five pine tree species

**DOI:** 10.1080/23802359.2019.1710587

**Published:** 2020-01-14

**Authors:** Qunfeng Luo, Zhangqi Yang, Yuanheng Feng, Jie Jia, Jianhui Tan, Peidong Yan

**Affiliations:** Guangxi Key Laboratory of Superior Timber Trees Resource Cultivation, Guangxi Institute of Forestry Science, Nanning, China

**Keywords:** Gymnosperm, chloroplast genome, codon bias, phylogenetic analysis

## Abstract

*Pinus* plants are the largest existing group of gymnosperms and one of the most highly differentiated taxa. Due to its huge ecological, economic, and scientific value, the genetic diversity and the relationship between the intraspecific evolution of *Pinus* plants have gained wide attention. In this study, the chloroplast genomes of several common pine trees in southwest and south China, including *P. massoniana* (masson pine), *P. yunnanensis* (yunnan pine), *P. latteri* (south asia pine), *P. crassicorticea* (la ya pine), and *P. elliottii* (slash pine), and entire cpDNA sequences were obtained. Characteristics including the structure, repeated sequence, and codon bias of the cpDNA for these five pine tree species were analyzed.

The *Pinus* is mainly distributed in the northern hemisphere and is divided into the subgenera *Pinus* and *Strobus*. Due to its huge ecological, economic, and scientific value, the genetic diversity and the relationship between the intraspecific evolution of *Pinus* plants have gained wide attention (Mardanov et al. 2008; Huang et al. 2014).

In this study, high-throughput sequencing technology was used to sequence the chloroplast genomes of several common pine trees, including *P. massoniana* (Gui GC833D), *P. yunnanensis* (Gui GC394E), *P. latteri* (Gui GC1382E), *P. crassicorticea* (Gui GC846E), and *P. elliottii* (Gui GC111E), with the GenBank accession codes: MH701846\MK135066\MK000550\MK105898\MK105897. All samples were collected from the germplasm resource bank of the Nanning Forestry Research Institute (N23°10′, E108°00′) located in Wuming County, Guangxi, China. DNA library construction and sequencing were performed using the Illumina HiSeq PE150 strategy. Using the published chloroplast genome sequence of *P. taeda* (loblolly pine) as a reference, chloroplast reads for five samples were extracted and analyzed (Besemer et al. 2001; Lohse et al. 2007; Luo et al. 2012). Among the five chloroplast genomes, *P. elliottii* had the largest chloroplast genome, with a total length of 119,876 bp; *P. latteri* had the smallest chloroplast genome, with a total length of 119,715 bp. The difference in GC content was within 0.1% among the five pine tree species (Figure 1).

**Figure 1.**
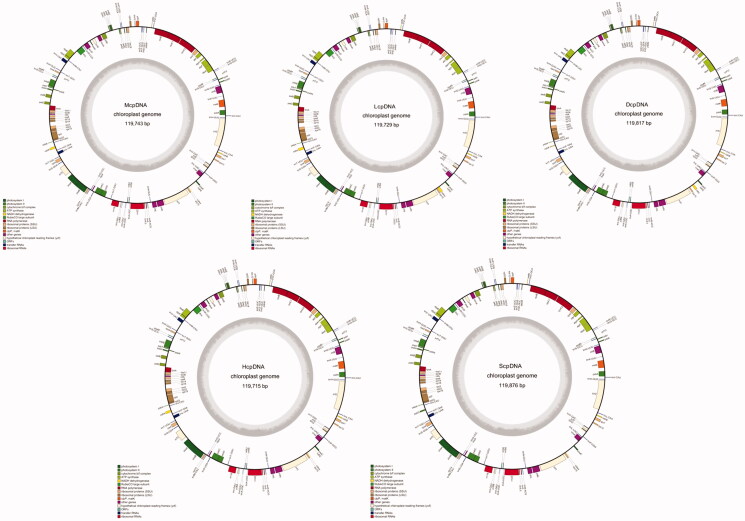
Gene maps of the chloroplast genomes.

The coding gene prediction showed that the number of coding genes was 48 in *P. massoniana* cpDNA and in *P. crassicorticea* cpDNA, and 47 in *P. yunnanensis* cpDNA, *P. latteri* cpDNA, and *P. elliottii* cpDNA. *P. latteri* had the longest average gene length (954 bp), and *P. yunnanensis* had the shortest (826 bp). *P. massoniana*, and *P. crassicorticea* had the highest ratio of gene length to whole genome length (37.59%), and *P. yunnanensis* had the smallest ratio (32.39%). Forthese protein-coding chloroplast genes, base A had the highest frequency at the first position of corresponding codons, whereas base T had the highest frequency at the second and third positions. Thus, the codons of chloroplast proteins prefer to end with A–– and T––. Except for *P. yunnanensis*, more than 70% of the codons in the other four pine tree species ended in A–– or T––. Based on the RSCU (relative synonymous codon usage, Xu et al. 2010) values of the chloroplast protein-coding genes in the five pine tree species, the results showed that these species had an adequate preference for codon use.

The chloroplast genomes of 16 *Pinus* plant species were subjected to sequence alignment to construct phylogenetic trees for the study of genetic geography. MAFFT v7.394 software was first used for sequence alignment (Kazutaka and Standley 2013), and the ML (maximum-likelihood) and BI (Bayesian inference) methods were then used to construct phylogenetic trees. The results indicated that the phylogenetic trees constructed by the two methods were virtually consistent (Figure 2).

**Figure 2.**
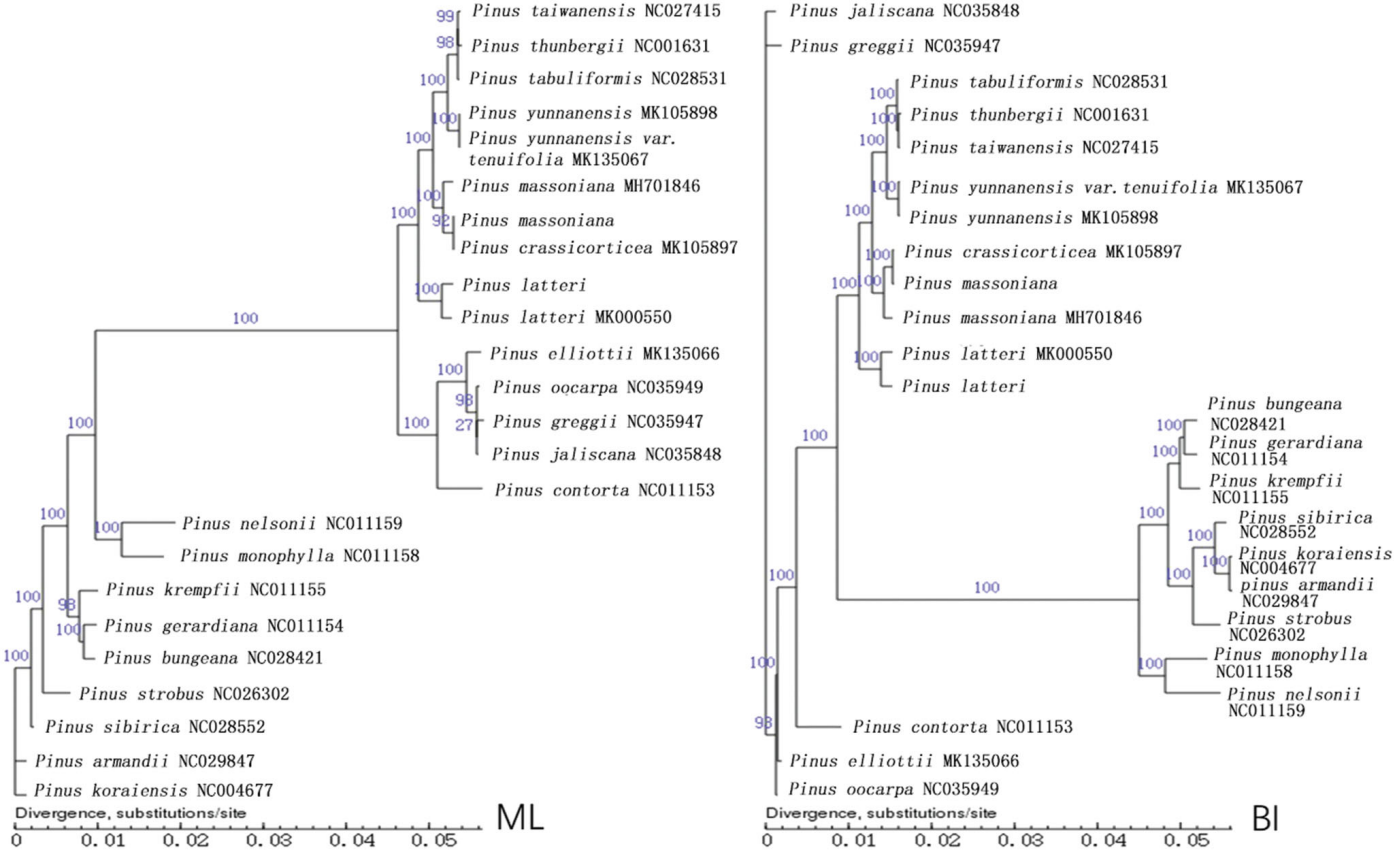
Clustering of 24 *Pinus* species based on complete chloroplast genome sequences using the maximum-likelihood (ML) and Bayesian Inference likelihood (BI) methods.
